# “WE CAN BE PART OF YOUR PLAN”: ADAPTING AND PILOTING A VA EMERGENCY PREPAREDNESS TOWN HALL FOR HOME HEALTH AGENCIES

**DOI:** 10.1093/geroni/igae098.2367

**Published:** 2024-12-31

**Authors:** Lauren Hall, Tamar Wyte-Lake, Emily Franzosa, Emily Solorzano, Aram Dobalian

**Affiliations:** James J Peters VA Medical Center, Bronx, New York, United States; U.S. Department of Veterans Affairs (VA), North Hills, California, United States; James J Peters VA Medical Center, Bronx, New York, United States; U.S. Department of Veterans Affairs (VA), Los Angeles, California, United States; U.S. Department of Veterans Affairs (VA), North Hills, California, United States

## Abstract

Home health agencies (HHAs) provide vital community-based care that helps older adults age in place. As climate-related disasters increase in frequency and intensity, HHA services become more critical. However, research shows HHAs are often under-resourced and disconnected from community-wide emergency planning efforts, threatening the continuity of services. As the nation’s largest integrated health system, the Veterans Health Administration (VHA) is uniquely positioned to support community HHAs serving veterans during disasters. We interviewed 19 key stakeholders (VA emergency managers, VA primary and community care staff, and administrators of contracted HHAs) from six VHA Medical Centers (VAMC) in a northeastern region (VISN 2) to generate strategies for VA to better support contracted home health providers. Participants offered innovative ideas including relatively low-resource suggestions and higher-level interventions (email groups, pocket emergency guides, staff training). Based on one site’s successful training for community providers of Medical Foster Home services, we worked with stakeholders to adapt and pilot a town hall and “resource fair” for home health agencies, scheduled for July 2024. Core sessions include guidance preparing emergency plans, an overview of VA benefits, and links to community resources. Stakeholders can also access 1-to-1 support on specific issues and offer suggestions for trainings VA can offer home health aides. Stakeholders identified potential challenges of town halls, including HHAs’ need to balance emergency preparedness with other priorities, and the broad geographical area which could limit attendance. This presentation will cover successes, challenges and lessons learned as we plan for scaling this effort at other sites.

